# Effect of 0.01% Atropine on Accommodation in Myopic Teenagers

**DOI:** 10.3389/fphar.2022.808440

**Published:** 2022-02-08

**Authors:** Huixia Li, Liying Zhang, Hong Tian, Song Zhang, Xueyan Zhang, Han Zhang, Yujing Chen, Wenping Qi, Xiaoying Wu, Hongmei Jiang, Hailong Yang, Yajun Yang, Lei Liu, Guisen Zhang

**Affiliations:** ^1^ Department of Retina, Inner Mongolia Chaoju Eye Hospital, Hohhot, China; ^2^ Department of Cataract, Ulanqab Chaoju Eye Hospital, Ulanqab, China; ^3^ Department of Cataract, Baotou Kunlun Chaoju Eye Hospital, Baotou, China; ^4^ Department of Graduate School, China Medical University, Shenyang, China; ^5^ Department of Cataract, Baotou Chaoju Eye Hospital, Baotou, China; ^6^ Department of Myopia, Chifeng Chaoju Eye Hospital, Chifeng, China; ^7^ Department of Ophthalmology, Guangdong Eye Institute, Guangdong Provincial People’s Hospital, Guangdong Academy of Medical Sciences, School of Medicine, South China University of Technology, Guangzhou, China; ^8^ Department of Ophthalmology, The First Affiliated Hospital of China Medical University, Shenyang, China

**Keywords:** accommodation, low-concentration atropine, myopia, ocular biometric components, axial length, spherical equivalent

## Abstract

**Purpose:** The purpose of the study is to evaluate the effects of 0.01% atropine eye drops on accommodative system parameters among teenagers with low myopia.

**Methods:** Ninety-five myopic teenagers [39 boys (8.69 ± 2.473) and 56 girls (8.54 ± 2.054) aged 5–17 years] with no history of eye disease were enrolled. Biometric and accommodative system parameters were evaluated before and at 1 week, 1 month, 3 months, and 6 months of 0.01% atropine eye drop instillation.

**Results:** Participants without accommodative demand at 6 months demonstrated insignificant changes after the atropine instillation (all *p* > 0.05). Nevertheless, there were significant differences in accommodative sensitivity, accommodative amplitude, accommodative responsiveness, and negative relative accommodation (NRA) at 3 months compared with baseline after atropine instillation (all *p* < 0.05). Except spherical equivalent refraction, cornea thickness, intraocular pressure, and axial length were stable after the 0.01% atropine instillation (all *p* > 0.05).

**Conclusion:** Morphologically, current measurements suggested that 0.01% atropine had favorable reduction of accommodation for childhood low myopia over a half-year period.

## Introduction

Myopia has become a significant global public health and socioeconomic problem (Xue et al., 2018). It has been predicted that 49.8% of the global population is predicted to be myopic, and 9.8% of the global population is predicted to be high myopic in 2050 (Holden et al., 2016). Most developed countries, especially among East Asia, have been currently faced with high prevalence of myopia as well as high myopia (Morgan et al., 2012). Several studies report that the occurrence and progression of myopia are related to accommodation (Holden et al., 2014; Jonas et al., 2021). Previously, atropine can be used for cycloplegia before optometry, and the treatment of amblyopia, iridocyclitis, and malignant glaucoma (Chen et al., 2021; Pineles et al., 2021). In recent years, this “old drugs” with new application was used to control myopia (Saw et al., 2002; Chia et al., 2016; Hieda et al., 2021). However, the mechanism of its use to control myopia is still unclear. The efficacy of atropine in controlling myopia is positively correlated with concentration (Chia et al., 2012; Chia et al., 2014). The higher the concentration, the better the myopia controlling effects, while the more obvious the adverse reactions caused by this drug (Pineles et al., 2017). Recent studies have shown that low-concentration atropine (0.01%) not only retains the effect of myopia control but also greatly reduces the incidence of adverse effects (Chia et al., 2016). However, long-term follow-up studies on the effects of atropine on accommodative parameters have not been evaluated comprehensively. Therefore, the current study used a nonrandomized before and after controlled design to observe the effects of 0.01% atropine on accommodative system parameters.

## Methods

### Subjects

Teenagers with low myopia were continuously enrolled from April 2019 to March 2021. This study followed the guidelines outlined in the Declaration of Helsinki and was approved by the Institutional Review Board (IRB) and research ethics committee of Hohhot Chaoju Eye Hospital. All parents/guardians of participating teenagers received a full explanation of the procedures and provided written informed consent. This clinical study registration number is ChiCTR2000034981.

### Inclusion Criteria

None of the participants had a history of eye disease, except for refractive errors, and all had a best-corrected visual acuity of 20/20 or better, with mydriatic spherical equivalent (SE) refractive error of −1.50 D to −0.75 D, astigmatism less than −1.00 D, and with normal intraocular pressure (<21 mmHg).

### Exclusion Criteria

We excluded subjects who were with any history of ocular disease, previous ocular surgery, or taking systemic medications that could affect accommodation.

### Refractive Status Check

All included subjects were treated with 1% atropine for cycloplegia, and a computerized automatic refractor (RC-4000, Tomey, Japan) and a retinoscope (YZ24, Suzhou Liuliu Vision Technology Co., Ltd., Suzhou, China) were used for objective flexion. For the detection of the optical state, the optometrist used a comprehensive refractor (DK-500, Japan Topcon Company) to perform subjective optometry to measure the final diopter the next day.

All included subjects underwent comprehensive ophthalmological examinations including best corrected visual acuity (BCVA), full optometric, noncontact tonometry, biometrics and slit lamp microscope, fundus, and accommodative function measurements before and at 1 week, 1 month, 3 months, and 6 months after 0.01% atropine eye-drop use. An experienced technician in each of the hospital carried out all the examinations. All subjects underwent low-concentration atropine (0.01%) eye drop instillation once a day.

### Accommodative Amplitude Measurement

Refractive errors were corrected with the best refractive compensation in place during the accommodative tests if teenagers were needed. The near vision card (Near Chart) at a distance of 40 cm was set according to “minus lens method,” so that the examinee could see the upper line of the optotype clearly. Minus and plus lenses with 0.25 D steps were increased subsequently until the first sustained blur.

### Accommodative Sensitivity Measurement

The participant wears a telescopic correction lens with ±2.00 D inversion shot, and looked at the Near Chart at 40 cm (the upper line of the best visual acuity mark), and first place +2.00 D in front of both eyes. It was reported immediately when the reader read clearly, and immediately reversed to −2.00 D, until the font was clear and then reversed again. Finally, the numbers of cycles completed within 1 min were recorded.

### Accommodative Response

The optometrist used a comprehensive refractometer (VT-10, TOPCON, Japan) on the basis of long-distance correction, and placed a 0.50 D cross column in front of the eyes of the participant. After that, the participant should fix the negative axis of the cross cylinder to 90° and the positive axis to 180°, respectively. Then the patient was asked to look at the fused cross cylinder through the cross cylinder to test the visual target. If the participant replied that the horizontal line was clearer than the vertical line, it reflected that the participant should gradually add a 0.25 D lens positive lens in front of the participant’s eyes until the subject could see the two lines as clearly as well. If the participant replied that the vertical line was clearer, it reflected advanced adjustment. The adjustment would be made by gradually adding a negative lens with a 0.25 D lens in front of the participant’s eyes, gradually, one by one, until the participant could see the two lines equally clearly.

### Negative Relative Accommodation and Positive Relative Accommodation

Measurements were set at a distance of 40 cm on the basis of distance correction of both eyes. For negative relative accommodation (NRA), the positive lens with +0.25 D was added one at a time and fixed in front of the eyes of the participant until the target was blurred to the subject. The total number of added lenses recorded is the NRA value. For positive relative accommodation (PRA), the negative lens was added one at a time in front of the eyes of the participant with −0.25 D until the target was blurred. The number of negatives added last is the value of PRA.

### Accommodation Convergence/Accommodation Ratio

Subjective binocular detection was first evaluated at a distance of 6 m, and then at a distance of 40 cm. A phoropter was used to measure horizontal occlusion, placing a 6 Δ base-up separation prism in front of the left eye to deviate the vertical oculomotor nerve, and then using a 12 Δ base-in separation prism to neutralize the horizontal oculomotor deviation in the front of the right eye until it induces diplopia. Then the number of prisms was reduced until the subject could just recover from the diplopia image. Three hidden slope measurements were obtained for each subject, and the average value was taken. The accommodation convergence/accommodation (AC/A) ratio was calculated using the heterophoria method.

### Statistical Methods

The SPSS 22.0 statistical software (SPSS Inc., Chicago, IL, USA) was used to analyze the data. Categorical variables were summarized by percentage. Numerical data were expressed as the mean ± standard deviation (SD) or median/interquartile range (IQR). Mann–Whitney U-test was used to test for difference between the right and left eyes. The nonparametric Wilcoxon matched-pairs signed-rank test was used to identify the difference among baseline and four different follow-ups (Tan et al., 2021). A *p*-value less than 0.05 was identified as statistically significant.

## Results

### Baseline Characteristics of the Participants

Ninety-five teenagers [39 boys (8.69 ± 2.473) and 56 girls (8.54 ± 2.054)] all underwent 0.01% atropine treatment, and no severe complications were observed during the follow-up period. The mean age of the participants was 8.60 ± 2.22 (range 5–17) years. Table 1 presents the baseline biometric and accommodation system parameters of both eyes before the instillation of 0.01% atropine eye drops. The cycloplegic refraction of the right eye ranged from −2.75 D to 0.25 D, and that of the left eye ranged from −2.25 D to 1.875 D; thus, there was no significant difference in refraction between the right and left eyes (*p* = 0.133). Furthermore, there was also no significant difference in cornea thickness, intraocular pressure, and axial length as well as accommodation parameters between the right and left eyes (all *p* > 0.05). Therefore, we present the findings of only the right eye.

**TABLE 1:** Baseline biometric parameters of both eyes before instillation of 0.01% atropine eye drops.

	Right eye		Left eye		*p*-Value
	Median	Range	Median	Range	
SE (D)	−0.75	[−2.75, 0.25]	−0.75	[−2.25, 1.875]	0.133
Cornea thickness (μm)	537	[464, 617]	541	[463, 614]	0.825
IOP (mmHg)	17	[10, 24]	17	[10, 25]	0.849
AL (mm)	23.66	[22.13, 27.89]	23.62	[22.02, 25.84]	0.454
Accommodative sensitivity (cpm/min)	6	[0, 15]	6.00	[0, 13]	0.990
Accommodative amplitude (D)	11.5	[0, 18]	12.00	[0, 20]	0.896
Accommodative responsiveness (D)	0.25	[−0.5, 1]	0.25	[−0.5, 1]	0.990
NRA (D)	2	[0.5, 3]	2.00	[0.5, 3]	0.990
PRA (D)	−2	[−5, 1.75]	−2	[−5, 1.75]	0.990
AC/A	3	[0, 6]	3.00	[0, 6]	0.990

Note. PRA, positive relative accommodation; NRA, negative relative accommodation; AC/A, accommodative convergence/accommodation ratio; IOP, intraocular pressure; AL, axial length; SE, spherical equivalent.

### Effects of 0.01% Atropine on Refraction and Biometric Parameters

**FIGURE 1:**
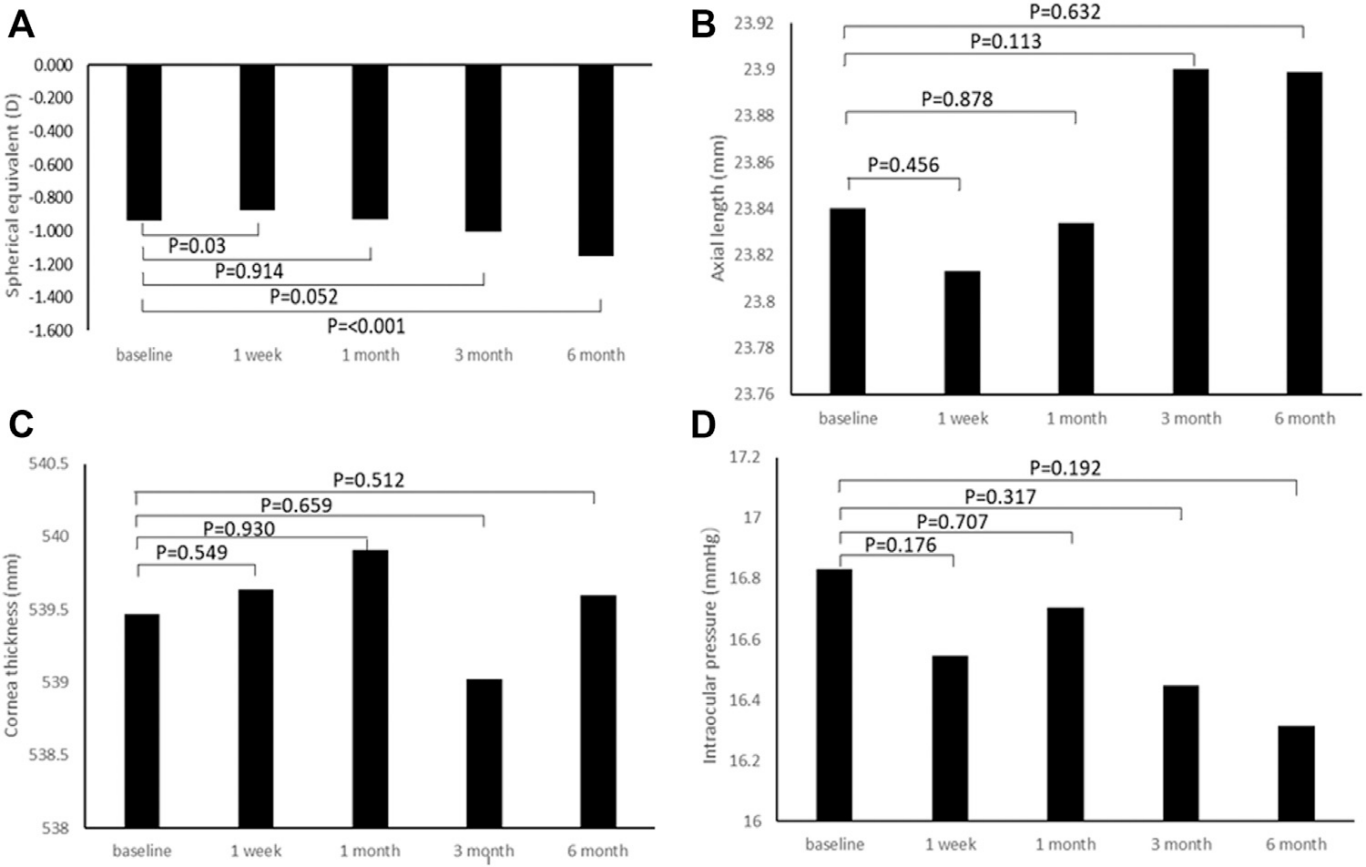
Change in biometric parameters after the instillation of 0.01% atropine eye drops. **(A)** Spherical equivalent. **(B)** Axial length. **(C)** Cornea thickness. **(D)** Intraocular pressure.

Figure 1A presents a comparison of SE in the relaxed state before and 1 week, 1, 3, and 6 months after instillation of 0.01% atropine eye drops. The SE was improved 1 week after atropine instillation than before in the relaxed state (*p* = 0.03). In contrast, there was no significant difference in the RE 1 and 3 months after atropine instillation compared with that before atropine instillation in the relaxed state, while it was significant in the RE 6 months after atropine instillation compared with before atropine instillation. Figures 1B–D show that there are no significant differences in AL, cornea thickness, and intraocular pressure (IOP) before and after atropine instillation (all *p* > 0.05).

### Effects of 0.01% Atropine on Accommodative System Parameters

**FIGURE 2:**
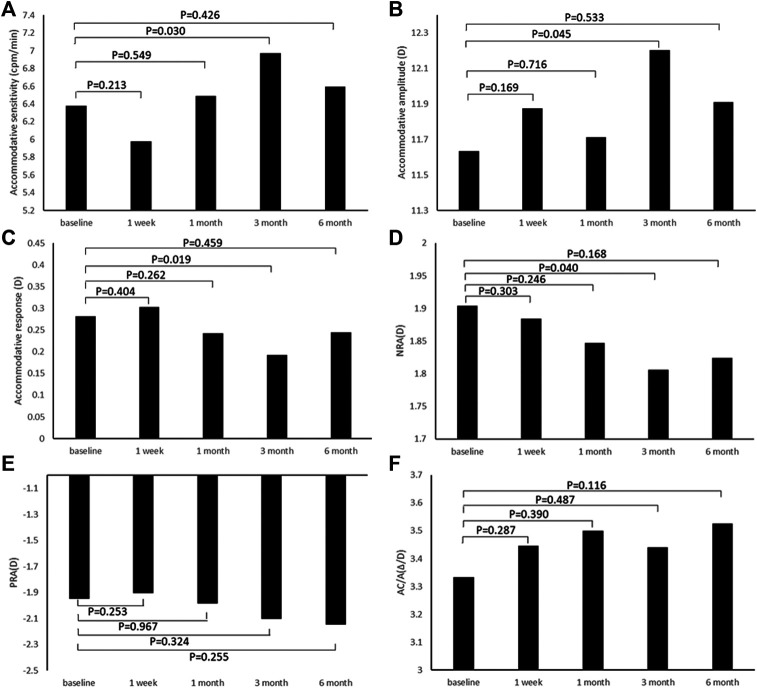
Change in accommodative system parameters after the instillation of 0.01% atropine eye drops. **(A)** Accommodative sensitivity. **(B)** Accommodative amplitude. **(C)** Accommodative response. **(D)** NRA, negative relative accommodation. **(E)** PRA, positive relative accommodation. **(F)** AC/A, accommodative convergence/accommodation ratio.

Figure 2A shows the accommodative sensitivity 1 week, 1 month, 3 months, and 6 months after the instillation of 0.01% atropine eye drops. Except for the 3 months, accommodative sensitivity, accommodative amplitude, accommodative responsiveness, and NRA did not change significantly at the three follow-ups after the instillation of 0.01% atropine eye drops compared with baseline, respectively (all *p* > 0.05, Figures 2B–D). However, the differences in positive relative accommodation (PRA) and AC/A value were insignificant before and after the instillation of 0.01% atropine eye drops (all *p* > 0.05, Figures 2E,F).

## Discussion

In order to prevent the outbreak of coronavirus disease 2019 (COVID-19), lockdown has caused many students to stay indoors in many countries, which may promote myopic occurrence and progression. With the increased near work and insufficient outdoor activities during lockdown, approximately 80% of outpatient visits in the pediatric ophthalmology department in March and April 2020 were for refractive errors (RE), of which 79% were for myopia (Jayadev et al., 2020). Recently, a prospective cross-sectional study, using school-based photoscreenings involving 123,535 Chinese children aged 6–13 years during the COVID-19 home confinement, has reported a significant myopic shift for children aged 6–8 years of approximately −0.3 D (Wang et al., 2021). These findings are consistent with the outcomes of another large-scale intervention research with 12-month follow-up on myopia development among 1,001,749 school children of 1,305 elementary and high schools. Here, myopia increased approximately 1.5 times from −0.23 D during the pre-COVID-19 to −0.343 D during the post-COVID-19 period (Toro et al., 2021). Moreover, even if the data are still controversial, besides myopic progression, accommodative dysfunction in children may be also on the rise due to digital device usage. Accommodation spasm and sudden-onset esotropia are important consequences.

To some extent, better vision is associated with better accommodative function (Chen et al., 2019). Previous studies have shown that low-concentration atropine effectively inhibits the progression of myopia and axial elongation (Azuara-Blanco et al., 2020; Polling et al., 2020; Walline et al., 2020). However, the effects of 0.01% atropine on accommodative system parameters are still unclear. Our prospective nonrandomized single-arm study revealed that 0.01% atropine had no effect on accommodative system parameters before and 6 months after instillation. Interestingly, accommodative sensitivity, accommodative amplitude, accommodative responsiveness, and NRA changed significantly at the 3 month follow-up after the instillation of 0.01% atropine eye drops compared with baseline, respectively.

To date, there are a few reports evaluating the effects of low-concentration atropine on the accommodative system. Previously meta-analysis revealed that patients receiving 0.01% atropine showed no significant changes in accommodative amplitude (WMD, −0.45 D; 95% CI = −1.80, 0.90; *p* = 0.51) over a 1-year period (Tsai et al., 2021), which is consistent with our findings. However, in another study, Caucasian children receiving a concentration of 0.05 and 0.01% atropine revealed that accommodation was decreased by −4.2 ± 3.8 D in 0.05% atropine-treated eyes, whereas 0.01% atropine induced hypoaccommodation of −0.05 ± 2.5 D (*p* < 0.01) (Joachimsen et al., 2021). There is a nonlinear dose–response relationship between atropine and accommodative amplitude (Tran et al., 2021). Herein, future study is needed to determine the concentration that provides maximal efficacy with tolerable effects on the accommodative system.

In the present study, compared with baseline, a significant reduction in SE after 0.01% atropine eye drop instillation was observed at 1 week. Although the changes in AL were not significant compared with baseline at 1 week, the decrease in AL was observed. This trend was consistent with the findings of Ye et al. (2021). These findings suggest that the improved effect of 0.01% atropine eye drop instillation on SE might be affected by the shortened AL. Recently, it has been reported that one of the effects of atropine is that it stimulates choroidal thickening (Chiang et al., 2020; Mathis et al., 2021; Ye et al., 2021). However, our findings were specifically created to have opposing effects at 6 months after atropine eye drop instillation.

Limitations of the present study should be acknowledged. Clinically, it is difficult to make adolescents receive 0.01% atropine intervention for 12 months, and our observation duration is 6 months. Herein, the follow-up period of the current study was not long enough to judge about the accommodative system parameter change. We did not have a healthy control group to observe the natural process of disease, which was a big limitation. Finally, we did not include the teenagers with moderate or high myopia because of the relatively small number of participants. Therefore, further randomized controlled trial should be performed to provide more evidence on the accommodation function after the instillation of atropine eye drops.

## Conclusion

Generally, our study observed the effects of 0.01% atropine eye drops on the accommodative parameters, and it is necessary to observe accommodative parameters at 3 months after the 0.01% atropine eye drop instillation. Our findings provided information for its clinical application for controlling the development of myopia in teenagers.

## Data Availability

The original contributions presented in the study are included in the article/Supplementary Material. Further inquiries can be directed to the corresponding authors.
